# Subject-specific kinematic analysis of a total ankle arthroplasty implant

**DOI:** 10.3389/fbioe.2026.1855705

**Published:** 2026-08-28

**Authors:** Vincenzo Leone, Antonio Mazzotti, Cesare Faldini, Simone Ottavio Zielli, Francesca Di Puccio, Marco Viceconti, Cristina Curreli

**Affiliations:** 1 Medical Technology Lab, IRCCS Istituto Ortopedico Rizzoli, Bologna, Italy; 2 1st Orthopaedic and Traumatologic Clinic, IRCCS Istituto Ortopedico Rizzoli, Bologna, Italy; 3 Department of Biomedical and Neuromotor Science (DIBINEM), University of Bologna, Bologna, Italy; 4 Department of Civil and Industrial Engineering, University of Pisa, Pisa, Italy; 5 Centre for Sport and Rehabilitative Medicine “Rehab & Sport”, University of Pisa, Pisa, Italy; 6 Department of Industrial Engineering, Alma Mater Studiorum - University of Bologna, Bologna, Italy

**Keywords:** CT-based model, FAR prosthesis, planetary gear mechanism, subject-specific kinematics, total ankle arthroplasty, 4 bar linkage

## Abstract

Total Ankle Arthroplasty (TAA) is a well-established surgical treatment for end-stage ankle arthritis, yet functional outcomes often remain inferior to those of the native joint. This may be because restoring the full physiological range of motion would likely require a custom-made surgery, in which both the prosthetic design and component positioning are defined on the basis of a patient-specific kinematic analysis. The aim of this study is to propose a subject-specific kinematic analysis of an ankle joint implanted with the FAR prosthesis and to investigate the compatibility between prosthesis-induced motion and the physiological motion of the healthy joint. A CT-based 3D model and a 4-bar linkage model were used to estimate the physiological path of the instantaneous centre of rotation during passive plantar–dorsiflexion, while the kinematic constraints of the FAR prosthesis were simultaneously analysed. A novel methodology was proposed, based on an analogy between the FAR implant design and a planetary gear mechanism, to reproduce the physiological kinematics of the natural joint with high fidelity. The results, in terms of overall plantar–dorsiflexion range of motion and ligament behaviour, suggested that the analysed TAA design was unable to fully reproduce the physiological kinematics of the healthy joint model for the subject and virtual surgery considered. Comparison with clinical data demonstrated general agreement in terms of range of motion and meniscal bearing displacement. Overall, the proposed methodology provides a framework for systematically analysing subject-specific ankle joint kinematics after implantation and suggests promising directions for future research.

## 1 Introduction

Total Ankle Arthroplasty (TAA) has emerged in the past decades as a viable alternative to ankle arthrodesis, offering the advantage of restoring a proper range of motion preserving a more physiological gait pattern (Dekker et al., 2018; Almutairi et al., 2023). Despite providing significant pain relief and functional improvement, clinical outcomes often remain inferior to those achieved with the native ankle (Teehan and Demetracopoulos, 2024; Yoshikawa et al., 2025). One of the main challenges is restoring the Range of Motion (ROM) of the joint, which is strongly linked to the difficulty of re-establishing the physiological ankle kinematics and, in particular, the natural trajectory of the joint axis of rotation (Leardini et al., 2014).

The need to improve TAA implant designs has motivated extensive research into the kinematic principles governing the native ankle, leading to the development of progressively more sophisticated biomechanical models of the joint. Seminal experimental studies by Leardini and colleagues demonstrated that specific ligament fibres, particularly the calcaneofibular and tibiocalcaneal ligaments, exhibit quasi-isometric behaviour during passive motion and function as mechanical guides that regulate talar movement relative to the tibia (Leardini et al., 1999a; Leardini et al., 1999b). This has led the same authors to propose a representation of the ankle joint using a single degree of freedom 4-bar linkage system governed by ligament constraints, capable of predicting the two-dimensional sagittal kinematics of the human ankle–subtalar joint complex under unloaded conditions (Leardini et al., 1999c). Building on this concept, several equivalent kinematic and kinetostatic models have been proposed to capture the three-dimensional and load-dependent behavior of the joint (Forlani et al., 2015; Di Gregorio et al., 2007; Sancisi et al., 2014). More advanced musculoskeletal, contact-aware, and ligament-aware models have been developed to account for muscle forces, joint contact, stability, and load transfer (Liu et al., 2024; Peng et al., 2023; Wang et al., 2024). These approaches extend beyond purely kinematic descriptions and enable the investigation of physiological function, ligament injuries, cartilage stresses, and implant mechanics. However, simple 4-bar linkage models remain extremely useful for understanding the fundamental kinematic constraints governing the tibiotalar plantar-dorsiflexion.

Replicating physiological joint kinematics in TAA depends on multiple factors, including implant design, precise bone cuts, proper implant alignment, and balanced ligament structure and soft tissue (Louwerens, 2015). Postoperative clinical data have shown that an increase in ROM, especially dorsiflexion, is associated with better patient-reported outcomes (Palma et al., 2024; Fletcher et al., 2024). Recent studies have also focused on the influence of the patient’s native varus/valgus deformity and joint-line height on the ROM (Fletcher et al., 2024; Tochigi et al., 2005). The latest advancements in TAA are moving toward the adoption of patient-specific instrumentation and custom-made surgery, with prosthetic designs capable of better replicating natural joint kinematics (Faldini et al., 2024; Mazzotti et al., 2024). Among total ankle replacement systems, the FAR (Adler Ortho, Cormano, Italy) and the BOX (Finsbury Orthopaedics Limited, Leatherhead, United Kingdom) implant offer potential advantages owing to their ligament-isometry–driven design (Bianchi et al., 2021; Giannini et al., 2011). The two implants share the same three-component prosthetic design, including a mobile-bearing meniscal element, whereas the tibial and talar components do not replicate the native anatomical geometry. The FAR implant represents an evolution of the original BOX design, incorporating additive-manufactured titanium components and advanced porous and coated surfaces to enhance osseointegration and wear resistance, while preserving the same biomechanical concept and articular geometry.

Few studies have investigated the kinematics of the BOX prosthetic components. Most of them used imaging techniques to assess the *in vivo* kinematics of the device by measuring the relative motion of the implant components during various motor tasks (Caravelli et al., 2024; Ingrosso et al., 2008). Cenni et al., (2012a) digitally measured geometrical parameters on radiographic images from a series of patients who underwent ankle replacement with the BOX implant and evaluated the range of ankle flexion, assessing the influence of component positioning over the mobility of the replaced joint. A similar investigation was later conducted through the digital reconstruction of the component’s *in vivo* kinematic behaviour using videofluoroscopy techniques (Cenni et al., 2012b). Also, a modeling study conducted by Reggiani et al. (2006) used a three-dimensional numerical model of the BOX implant to assess its performance during functional activities. The results showed that simulated passive plantar–dorsiflexion reproduced kinematic patterns consistent with those predicted by the analytical 4-bar linkage model underlying the prosthesis design.

Although these studies provide valuable insights into the motion of the implanted joint, limited knowledge exists regarding how this motion compares with physiological ankle kinematics. In particular, methods capable of quantitatively assessing the extent to which implant design reproduces physiological joint mechanics on a patient-specific basis remain insufficiently developed in the current literature. The aim of this study is to fill this gap by proposing a biomechanical analysis of an ankle joint model obtained from a healthy patient, in which a TAA procedure using the FAR implant was simulated, investigating the compatibility between the theoretical motion allowed by the implanted ankle joint and the physiological motion of the healthy joint.

## 2 Materials and methods

**FIGURE 1:**
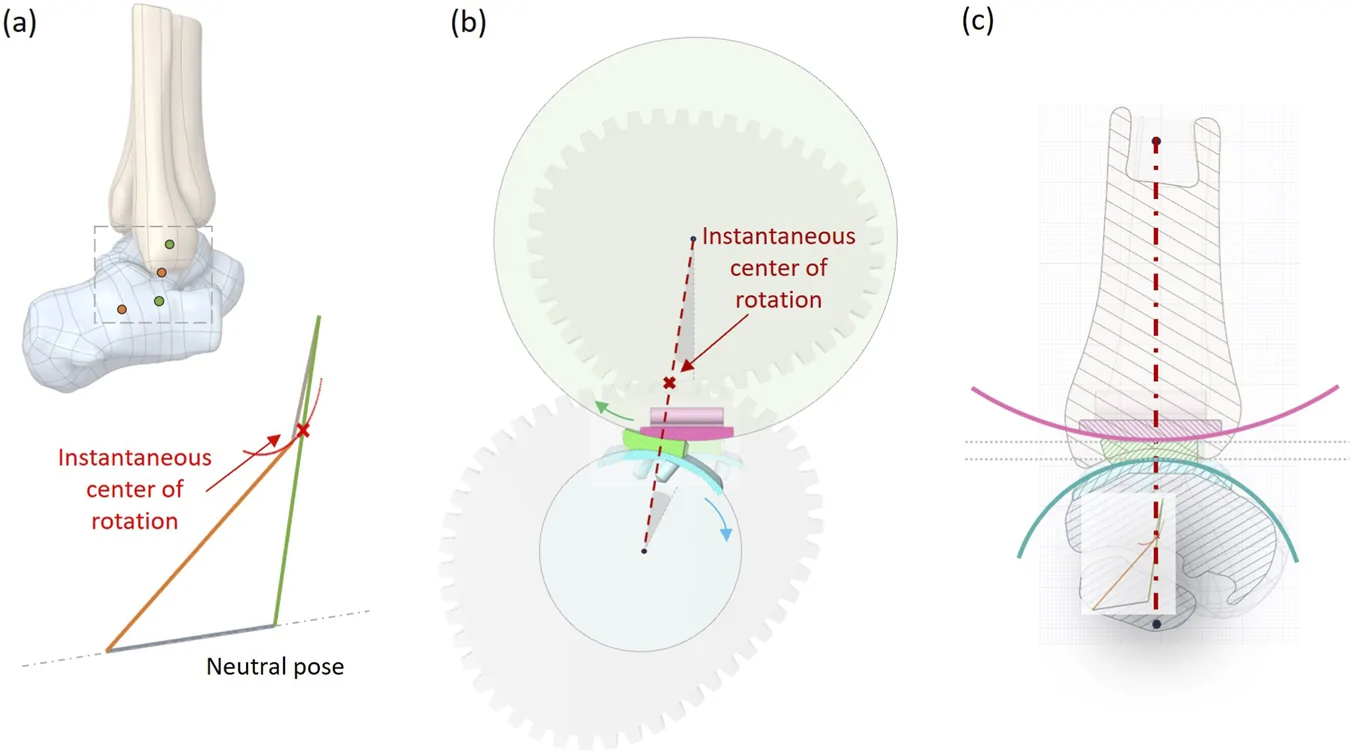
Different steps of the analysis performed to investigate the subject-specific kinematics of an ankle joint implanted with the FAR prosthesis: **(a)** 3D model showing the anatomical landmarks used to identify the joints of a 4-bar planar linkage, yielding the physiological path of the instantaneous centre of rotation; **(b)** FAR implant and kinematic mechanism with its instantaneous centre of rotation; **(c)** kinematic compatibility between the motion allowed by the implanted ankle joint and the physiological motion of the healthy joint.

A subject-specific kinematic analysis of an implanted ankle joint was performed considering different steps illustrated in Figure 1.

A 3D model of the ankle joint was first obtained from a CT scan capturing the neutral pose of a right foot and then used to construct a subject-specific 4-bar planar linkage model based on a well-established single-degree-of-freedom system during passive motion (Leardini et al., 1999b; Section 2.1). This model allows estimation of the physiological path of the instantaneous centre of rotation (CoR) of the talus–calcaneus complex relative to the tibia–fibula complex, over a given range of plantar–dorsiflexion angles (Section 2.2; Figure 1a).

Simultaneously, a systematic analysis of the FAR prosthesis design was conducted to identify and characterize the allowed relative motion between the implant components (Section 2.3; Figure 1b). Specific observations suggested a method for estimating the kinematics of the implant and the instantaneous CoR of the talar component relative to the tibial one. This method is based on an analogy between the FAR implant design, considered as a planar system with two degrees of freedom (DoFs), and a planetary gear mechanism (Section 2.4).

Finally, a virtual surgery was carried out, and information about the physiological path of the instantaneous centre of rotation of the talus–calcaneus complex relative to the tibia–fibula complex was considered with respect to the implant position and its kinematic model (Figure 1c). In particular, the compatibility between the motions allowed by the ankle prosthesis and the physiological motion of the healthy joint was analysed (Section 2.5).

### 2.1 Three dimensional model of the ankle joint

**FIGURE 2:**
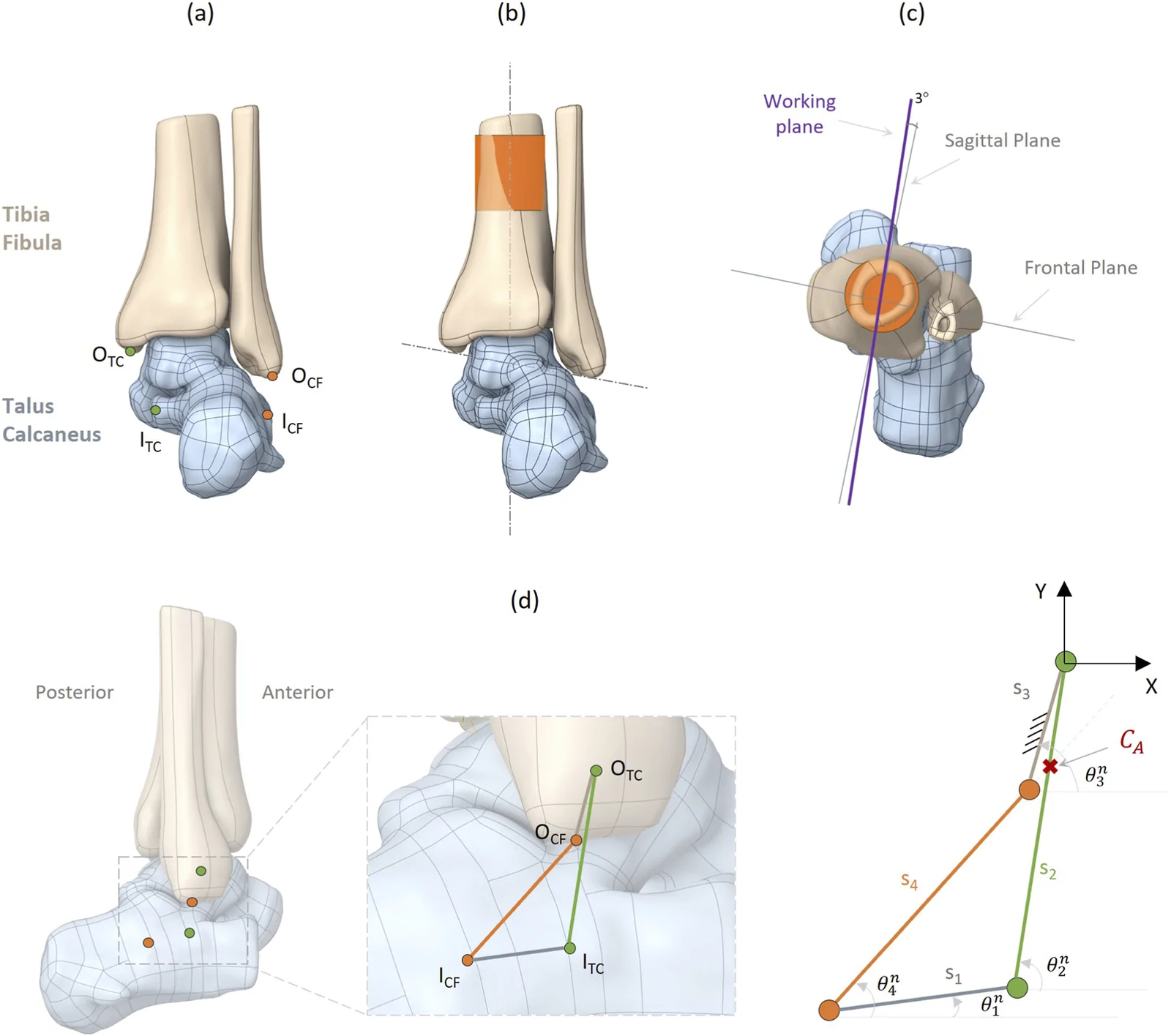
**(a)** Posterior view of the solid model, including the tibia–fibula and talus–calcaneus complex, with anatomical landmarks indicating the origin and insertion of the two ligaments. **(b)** Line passing through the malleolar tips and a cylinder (shown in orange) fitted in the tibial diaphysis, aligned with the vertical axis of the tibia. **(c)** Transverse plane view showing the frontal, sagittal, and working planes. **(d)** Subject-specific 4-bar linkage model and instantaneous centre of rotation of the mobile segment $s_{1}$ with respect to the fixed segment $s_{3}$ in the neutral pose.

A 3D model of the ankle joint was first obtained from a CT scan that captured the neutral pose of the right foot and ankle bones of a healthy subject with no prior history of injury to the analysed foot and ankle, under bipodal weight-bearing conditions. The software 3D Slicer was used to segment the distal portion of both tibia and fibula and the talus-calcaneus complex. The faceted volumes of the identified bone segments were then imported into Ansys SpaceClaim 2023 R2 (ANSYS Inc., United States) to obtain solid models and identify anatomical landmarks corresponding to the origin and insertion of the calcaneofibular ligament (O_CF_ and I_CF_, respectively) and the tibiocalcaneal ligament (O_TC_ and I_TC_, respectively). This procedure was assisted by a surgeon, guided by anatomical essays (Dalmau-Pastor et al., 2024; Golanó et al., 2010) and performed with the best effort to identify the landmarks as accurately as possible. An anatomical reference frame was introduced following the procedure prescribed in the study proposed by Leardini et al. (1999b). The malleolar tips were identified as the most distal points of the tibia and fibula. A cylinder was then fitted in the available portion of the tibial diaphysis and assumed to be aligned with its vertical axis. The plane passing through the two tips and parallel to the cylinder axis was defined as the frontal plane. The sagittal plane was assumed to pass through the vertical axis of the tibia and orthogonal to the frontal plane. As indicated in (Leardini et al., 1999b), a working plane was obtained by rotating the sagittal plane 3° anticlockwise around the axis formed by the intersection of the sagittal and frontal planes, as viewed from a superior position. The transverse plane was defined as orthogonal to the vertical axis of the tibia. The solid model, virtual landmarks and working plane are shown in Figures 2a–c.

### 2.2 4-bar linkage model

The subject-specific 4-bar planar linkage model, characterised by a single DoF, was constructed by projecting the palpated anatomical landmarks O_CF_, I_CF_, O_TC_ and I_TC_, onto the working plane and connecting them with two segments (O_CF_I_CF_ and O_TC_I_TC_) representing the ligaments, which were considered non-deformable. This assumption is supported by previous observations of the ligament’s behaviour under passive motion conditions (Leardini et al., 1999a). Accordingly, these ligaments were selected as two links of the ankle 4 bar linkage model, while the remaining two links corresponded to the tibia-fibula and talus-calcaneus complexes, modelled as rigid segments connecting the origins and insertions of the ligaments (I_TC_I_CF_ and O_TC_O_CF_, respectively) (Figure 2d). Within this model, the effect of contact between the articular surfaces is represented implicitly through the resulting kinematics rather than being modelled as an additional independent constraint. The model predictions showed good agreement with experimental measurements, supporting the validity of the model assumptions (Leardini et al., 1999c).

The geometry of the linkage was defined in the neutral pose (NP), and the lengths and orientations of each segment s_*i*_ of the 4-bar linkage model are reported in Table 1. A fixed reference frame was defined with its origin located at O_TC_ and its *y*-axis oriented parallel to the tibial shaft. Segment orientations are expressed as angles measured from the *x*-axis.

**TABLE 1:** Four-bar linkage configuration in the neutral pose (i.e., 0° of plantar-dorsiflexion angle), including segment lengths and orientations $\theta_{i}^{NP}$ with respect to the *x*-axis of the fixed reference frame. In the first column, *i* denotes the segment label.

*i*	Anatomical reference	*s*_*i*_ (mm)	$\theta_{i}^{NP}$ (°)
1	Talus-calcaneus complex	13.93	8.35
2	Tibiocalcaneal ligament	24.56	81.48
3	Tibia-Fibula complex	10.12	77.51
4	Calcaneofibular ligament	22.41	47.17

Since the analysis focused on the relative motion between the talar and tibial implant components, the tibia-fibula complex (segment *s*_*3*_) was treated as ground link, with $\theta_{3}$ assumed constant ( $\theta_{3}^{NP}=\theta_{3}$ ). The angle $\theta_{1}$ , defining the orientation of talus-calcaneus complex (segment *s*_*1*_) was chosen as the independent generalized coordinate. As the mechanism has a single degree of freedom, the configuration of the entire linkage is uniquely determined by the value of *θ*_1_, according to the procedure described below. Specifically, given the vector closure equation (Equation 1)

$$\vec{I_{CF}O_{CF}}+\vec{O_{CF}O_{TC}}=\vec{I_{CF}I_{TC}}+\vec{I_{TC}O_{TC}},$$ (1)

a set of two scalar equations can be obtained allowed unknown angles $\theta_{2}$ and $\theta_{4}$ to be determined as functions of $\theta_{1}$ :

$$s_{4}\;\cos\left(\theta_{4}\right)+s_{3}\;\cos\left(\theta_{3}\right)=s_{1}\;\cos\left(\theta_{1}\right)+s_{2}\cos\left(\theta_{2}\right)$$ (2)

$$s_{4}\;\sin\left(\theta_{4}\right)+s_{3}\;\sin\left(\theta_{3}\right)=s_{1}\;\sin\left(\theta_{1}\right)+s_{2}\;\sin\left(\theta_{2}\right)$$ (3)

The linkage configurations were evaluated across the physiological range of motion of the ankle plantarflexion-dorsiflexion angle $\theta_{PDF}$ , ranging from −40° in plantarflexion to 15° in dorsiflexion according to values reported in the literature (Kakkar and Siddique, 2011), (Lundberg et al., 1989). Such plantar-dorsiflexion angle $\theta_{PDF}$ is related to the deviation of the talus-calcaneus segment orientation $\theta_{1}$ from its orientation in the neutral pose, as expressed in Equation 4:

$$\theta_{PDF}=\theta_{1}-\theta_{1}^{NP}$$ (4)

An *ad hoc* Matlab script was developed to numerically solve Equations 2 and 3 using a nonlinear solver provided in the Optimisation Toolbox of MATLAB R2024a (MathWorks Inc, Natick, Massachusetts). For each of the $\bar{n}=100$ equally spaced values of $\theta_{PDF}$ spanning the investigated range, the corresponding values of $\theta_{2}$ and $\theta_{4}$ were computed.

Once the linkage configurations were determined, the instantaneous centre of rotation of the mobile segment $s_{1}$ with respect to the fixed segment $s_{3}$ (labelled $C_{A}$ in (Figure 2d) was identified as the intersection point of the lines defined by the two ligaments. Computing this intersection for each of the n configurations into which the plantar-dorsiflexion motion was discretized yielded to two discrete curves representing the path of $C_{A}$ in the fixed and in the mobile reference frame, (the latter rigidly attached to $s_{1}$ ). The curves correspond to the fixed and mobile centrodes, respectively (Uicker et al., 2017). The relative motion of the talar component with respect to the tibial component is completely characterised by the rolling of the mobile centrode over the fixed one.

### 2.3 FAR implant and allowed motions

**FIGURE 3:**
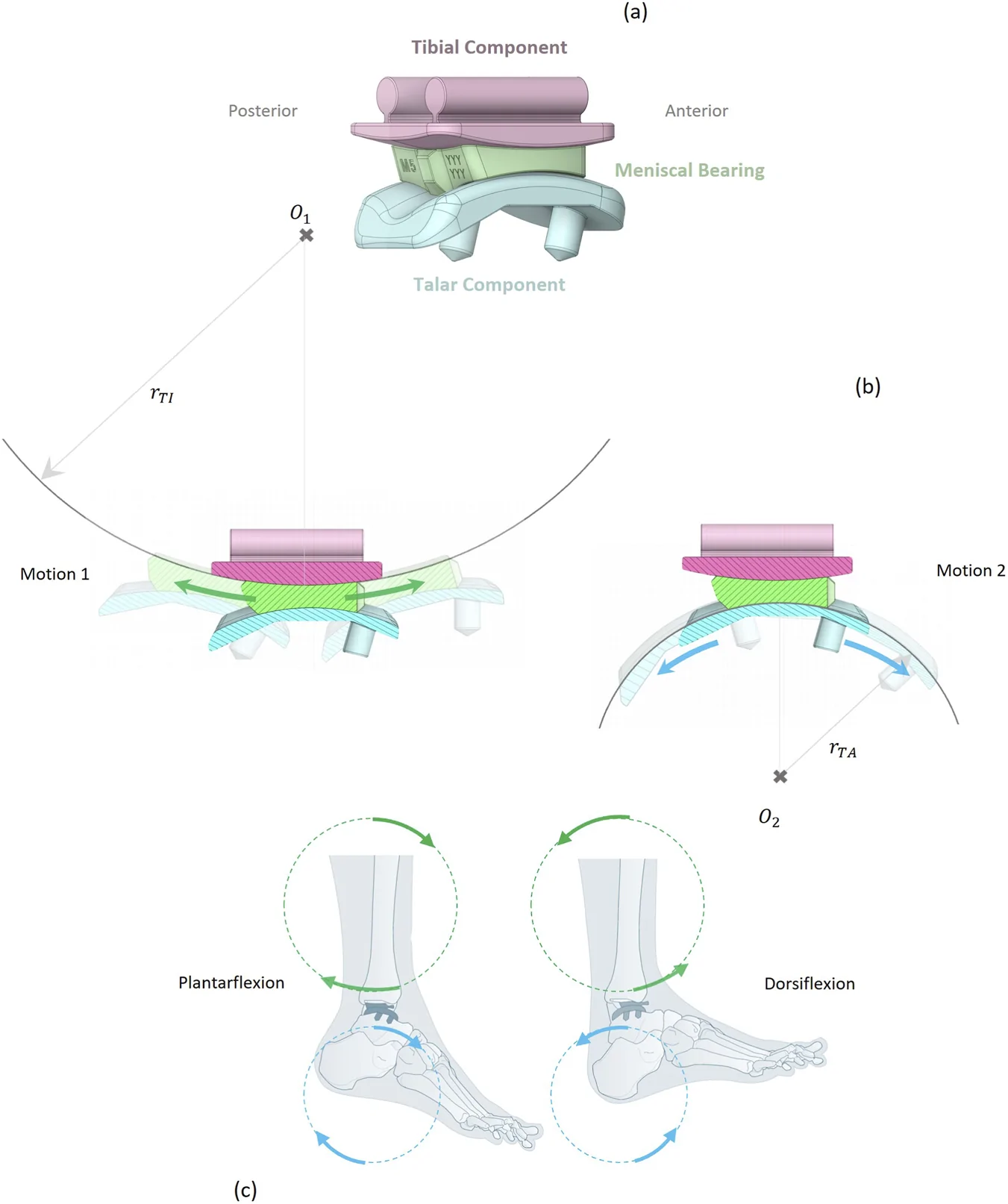
**(a)** The three components of the FAR implant; **(b)** simplified planar representation of Motion 1 (left) and Motion 2 (right), assuming the tibial component is fixed; **(c)** plantarflexion and dorsiflexion of the ankle, with rotations in the same direction.

The FAR total ankle replacement (Adler Ortho, Cormano, Italy) prosthesis was considered in this study. Its design shares geometrical features with the BOX (Finsbury Orthopaedics Limited, Leatherhead, United Kingdom), which was developed to restore the ligamentous role of stabilizing and guiding ankle motion (Leardini et al., 2004). The FAR implant consists of three fully congruent parts (Figure 3a): the tibial and talar components, made of titanium alloy and rigidly fixed to the respective bones and a meniscal bearing insert, made of ultra-high-molecular-weight-polyethylene. This plastic insert is designed to ensure fully congruent contact with both the tibial and talar components throughout the range of motion. The interface between the tibial component and the meniscus is a portion of a sphere, allowing for inversion-eversion, plantar-dorsiflexion and internal-external rotation of the foot. The interface between the talar component and the meniscus, which allows for only plantar-dorsiflexion, is a complex surface with a symmetry relative to the sagittal midplane; in this plane, its profile corresponds to a portion of a circle. Contact surfaces are designed in such a way that the line connecting the centres of the two arcs passes through the meniscal bearing at its minimum thickness in the sagittal midplane. The symmetry properties of the prosthesis design allows for a simplified planar representation, in which the three components are depicted by their section in the sagittal midplane (Figure 3b). This simplification facilitates the identification of the possible movements that the system can perform. Assuming the tibial component is fixed, the meniscal bearing can rotate rigidly about the centre of the tibial arc $O_{1}$ , along a circumference of radius $r_{TI}$ (Motion1). Additionally, the talar component can rotate relative to the meniscal bearing about the centre of the talar arc, $O_{2}$ , along a circumference of radius $r_{TA}$ (Motion 2) (Figure 3b). Furthermore, clinical evidence indicates that Motion 1 and Motion 2 are coupled when the prosthesis is implanted, such that both rotations have the same direction (Giannini et al., 2011; Leardini et al., 2004; Figure 3c). This means that when the plastic insert rotates clockwise around the tibial arc centre (displacement in the posterior direction), the talar component rotates clockwise around its arc centre (plantarflexion), with an additional translation dictated by its congruence with the insert. For either of these motions - or a combination of them–to occur, it is essential that the centre of the talar arc lies on a circumference positioned in the centre of tibial arc and with radius equal to the sum of talar radius, tibial radius and insert minimum thickness.

### 2.4 FAR kinematic mechanism

**FIGURE 4:**
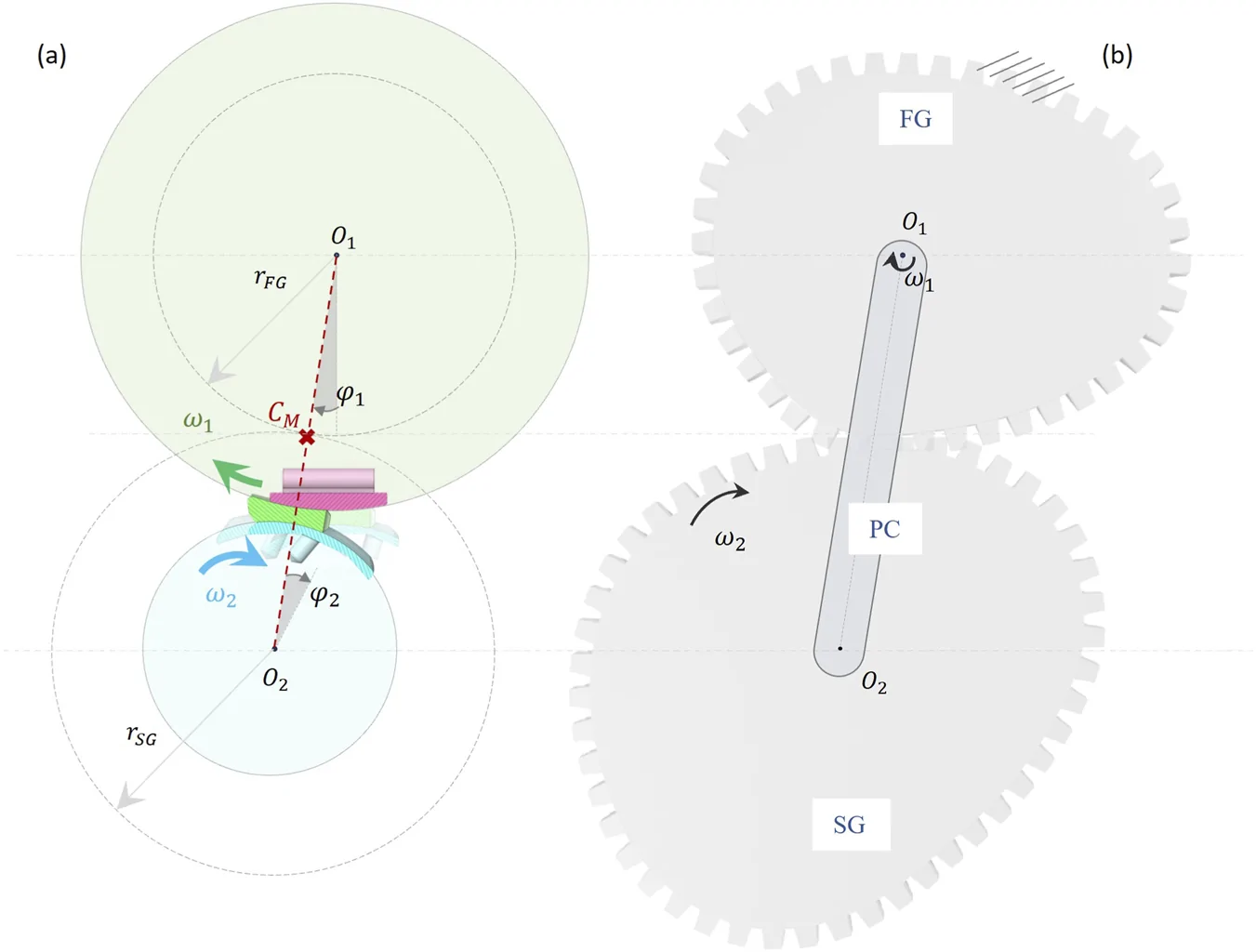
Articulated system in planar motion with two degrees of freedom, representing the kinematic of the FAR implant **(a)** and its analogy with the planetary gear mechanism **(b)**. In the neutral configuration, $\varphi_{1}$ = $\varphi_{2}$ = 0. The instantaneous centre of rotation of the talar component relative to the tibial one, $C_{M}$ , is shown for illustrative purposes only and is not intended to represent real configurations.

The 2D kinematics of the FAR implant were modelled as a planar system with 2 DoFs. The first degree of freedom, represented by the coordinate $\varphi_{1}$ , describes the rotation of the meniscal bearing relative to the tibial component about $O_{1}$ (Motion 1) whereas the second degree of freedom, represented by the coordinate $\varphi_{2}$ , describes the rotation of the talar component relative to the bearing about $O_{2}$ (Motion 2), as depicted in Figure 4a. As already reported, both rotations occur about parallel axes in the same direction and the motion of the talus relative to the tibia is given by a combination of Motion 1 and Motion 2. In particular, the angular velocities of the bearing and the talus relative to the tibia, denoted as $\omega_{1}$ and $\omega_{2}$ respectively, are given by Equation 5

$$\omega_{1}=\dot{\varphi_{1}}\;k\;\omega_{2}=\left(\dot{\varphi_{1}}+{\dot{\varphi }}_{2}\right)\;k$$ (5)

where ***k*** is the unit vector of the rotation axes, orthogonal to the working plane, according to the right-hand role. In this case, $O_{1}$ and $O_{2}$ are the instantaneous centres of rotation of the meniscal bearing relative to the tibial and of the talar component, respectively. According to Aronhold-Kennedy’s theorem (Uicker et al., 2017), the instantaneous centre of rotation of the talar component relative to the tibial one, $C_{M}$ , is expected to lie within the segment $O_{1}O_{2}$ (Figure 3a). The exact location of $C_{M}$ between $O_{1}$ and $O_{2}$ depends on the configuration and on the values of $\dot{\varphi_{1}}$ and ${\dot{\varphi }}_{2}$ , yielding different ratios of $O_{1}C_{M}$ / $O_{2}C_{M}$ .

Due to the clinical evidence discussed above, Motion 1 and Motion 2 were assumed to be coupled, such that $\varphi_{2}$ is uniquely determined by $\varphi_{1}$ (i.e., $\varphi_{2}$ = $\varphi_{2}\left(\varphi_{1}\right)$ ). As a result, the 2 DoFs system is reduced to an equivalent mechanism with a single DoF.

Considering these kinematic features, the FAR implant system may be represented as a non-circular planetary gear mechanism consisting of a fixed gear (FG), a satellite gear (SG), and a planet carrier (PC), as shown in Figure 4b. The centres of the FG and SG are located at $O_{1}$ and $O_{2}$ , respectively, while their instantaneous pitch radii are given by $O_{1}C_{M}$ and $O_{2}C_{M}$ . The pitch curves of the two gears are assumed to be in contact at the previously identified null-velocity point $C_{M}$ (Fuentes-Aznar et al., 2009). Planet carrier PC rotation occur with $\omega_{1}$ angular velocity around $O_{1}$ , representing the motion of the insert. Similarly, the gear SG rotates with angular velocity $\omega_{2}$ , corresponding to the absolute rotation of the talar component. This representation enables the computation of the gear ratio according to the Willis’ theory (Fuentes-Aznar et al., 2009). The planetary gear is first analysed in the reference frame of the carrier PC , so that the system becomes an ordinary gear with fixed axes of rotation. In this configuration, the instantaneous ordinary gear ratio $\tau_{0}$ is defined as reported in Equation 6:

$$\tau_{0}=\frac{\left(\omega_{2}-\omega_{1}\right)\cdot k}{\left(\omega_{FG}-\omega_{1}\right)\cdot k}=\frac{\omega_{SG\_PC}\;}{\omega_{FG\_PC}}=\frac{{\dot{\varphi }}_{2}\;}{-\dot{\varphi_{1}}}=-\frac{O_{1}C_{M}}{O_{2}C_{M}}$$ (6)

having considered $\omega_{FG}=0$ and that in $C_{M}$ , $O_{1}C_{M}\;\dot{\varphi_{1}}$ = $O_{2}C_{M}\;{\dot{\varphi }}_{2}$ .

Defining the instantaneous planetary gear ratio τ as the ratio between $\omega_{2}\cdot k$ and $\omega_{1}\cdot k$ , i.e.,

$$\tau =\frac{\omega_{2}\cdot k}{\omega_{1}\cdot k}=\frac{\dot{\varphi_{1}}+{\dot{\varphi }}_{2}\;}{\;\dot{\varphi_{1}}}$$ (7)

and comparing the latter with Equation 6, the following equation (Equation 8) can be achieved

$$\tau_{0}=-\tau +1$$ (8)

from which the instantaneous planetary gear ratio τ is expressed as function of the location of $C_{M}$ according to Equation 9

$$\tau =-\tau_{0}+1=-\frac{O_{1}C_{M}}{O_{2}C_{M}}+1=\frac{O_{1}O_{2}}{O_{2}C_{M}}$$ (9)

since $O_{1}O_{2}$ is constant. It can be observed that the angle ( $\varphi_{1}+\varphi_{2}$ ) defines the orientation of the talar component with respect to its neutral configuration, just as the plantar-dorsiflexion angle $\theta_{PDF}$ (Section 2.2) defined the deviation from the neutral orientation of the talus-calcaneus segment. It can be deduced that:

$$\varphi_{1}+\varphi_{2}=\varphi_{PDF}.$$ (10)

Combining Equations 7 and 10 the following relationship providing the plantar-dorsiflexion angle by integrating the gear ratio is obtained

$$\tau =\frac{\dot{\varphi_{1}}+{\dot{\varphi }}_{2}\;}{\;\dot{\varphi_{1}}}=\frac{{\dot{\varphi }}_{PDF}\;}{\;\dot{\varphi_{1}}}=\frac{d\;\varphi_{PDF}}{d\;\varphi_{1}}$$

$$\varphi_{PDF}=\int_{0}^{\varphi_{1}}\tau \;d\varphi_{1}.$$ (11)

### 2.5 Kinematic analysis of the implanted ankle joint

A virtual surgery implantation of the prosthesis into the subject-specific bone geometry (Section 2.1) was performed using Ansys Spaceclaim 2023 R2 (ANSYS Inc, United States) under the supervision of surgeons with experience in more than 300 foot and ankle procedures, following the standards described in the literature for the correct positioning of this type of device. The medium-sized prosthesis with a 5 mm thick insert was selected after inspection of the trochlea of the subject. The prosthesis components were aligned in the neutral configuration as described by the manufacturer, ensuring that the sagittal midsection of the implant coincided with the working plane in which the 4-bar linkage model had previously been constructed, and that the flat superior surface of the tibial component was orthogonal to the vertical axis of the tibia. Proximal-distal positioning was defined according to the surgical procedure described by Dalmau-Pastor et al. (2024). Antero-posterior positioning of the implant was chosen to achieve vertical alignment of the centre of the tibial arc $O_{1}$ , the centre of talar arc $O_{2}$ , and the crossing point of the ligaments, $C_{A}$ , in the neutral configuration (Leardini et al., 2004).

In order to assess the compatibility between the motions allowed by the ankle prosthesis and the physiological kinematics of the healthy joint, it was assumed that Motions 1 and 2 should occur such that the fixed and mobile centrodes of the talar component (SG) closely approximate those of the segment $s_{1}$ in the 4-bar linkage model, throughout the passive plantar-dorsiflexion motion considered in this study (Section 3.1). This implies that, in each configuration, $C_{A}$ and $C_{M}$ should be almost overlapped.

For each of the $\bar{n}=$ 100 points defining the centrode curve, obtained as described in Section 2.2, the corresponding values of the instantaneous planetary gear ratio $\tau^{n}$ and ${\varphi_{1}}^{n}$ (with $1\leq n\leq \bar{n}$ ) were then calculated moving from the predetermined coordinates of $C_{A}$ in a reference frame with origin in $O_{1}$ :

$$\vec{O_{1}C_{A}^{n}}=\left({x_{C_{A}}}^{n},{y_{C_{A}}}^{n}\right)\;\to {{\;\varphi }_{1}}^{n}={\text{tg}}^{-1}\;\frac{{x_{C_{A}}}^{n}}{{y_{C_{A}}}^{n}}$$

$$\vec{O_{1}O_{2}^{n}}=\vec{O_{1}C_{A}^{n}}\;\frac{O_{1}O_{2}}{O_{1}C_{A}^{n}}\;\to \;\vec{O_{2}C_{A}^{n}}=\vec{O_{1}O_{2}}\left({\varphi_{1}}^{n}\right)-\vec{{O_{1}C_{A}}^{n}}$$

$$\tau^{n}=\frac{O_{1}O_{2}}{O_{2}{C_{A}}^{n}};$$ (12)

The obtained discrete gear ratio curve was interpolated to obtain a polynomial function in $\varphi_{1}.\;A$ ninth-degree polynomial was selected to achieve the highest accuracy in fitting the computed data. According to Equation 11, the function was subsequently integrated to estimate the plantarflexion-dorsiflexion angle, as reported in Equation 13.

$${\varphi_{PDF\;}}^{n}=\int_{0}^{{\varphi_{1}}^{n}}\tau \left(\varphi_{1}\right)d\varphi_{1}$$ (13)

To reproduce the physiological kinematics of the ankle joint as faithfully as possible, the estimated ${\varphi_{PDF}}^{n}$ should not differ from the ${\theta_{PDF}}^{n}$ value corresponding to $C_{A}^{n}$ .

Additionally, the overall excursion of the plantar-dorsiflexion Δ $\varphi_{PDF}$ was evaluated over the interval of $\varphi_{1}$ (from ${\varphi_{1}}^{1}$ to ${\varphi_{1}}^{100}$ ), thereby yielding motion of the implanted ankle joint.

The determination of the two parameters $\varphi_{1}$ and $\varphi_{PDF}$ enabled a systematic description of the relative motion of the prosthetic components.

### 2.6 Quantities of interest

**FIGURE 5:**
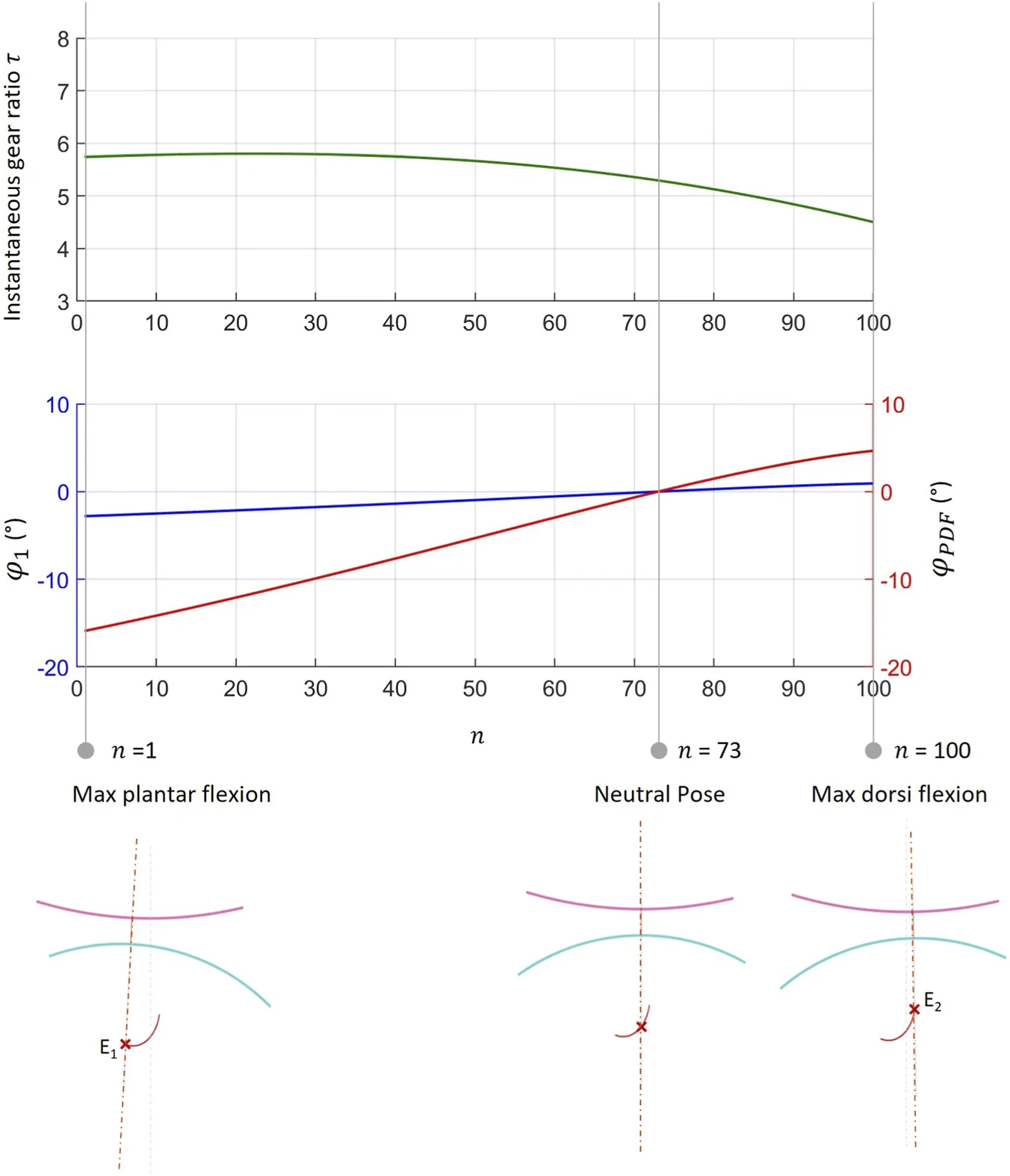
Trend of the instantaneous gear ratio τ , and of the angles $\varphi_{1}$ and $\varphi_{PDF}\;.$ Configurations corresponding to maximum plantar flexion, neutral position, and maximum dorsiflexion are also highlighted.

The kinematics of the implanted ankle joint was investigated considering the following quantities of interest:

- The range of variation of $\varphi_{1}$ (Δ $\varphi_{1}$ ), defined as the angle E_1_ ${Ô}_{1}$ E_2_, where E_1_ and E_2_ are the extreme points of the path of $C_{A}$ , as obtained from the 4-bar linkage model (Figure 5). This angle represents the rotation of the meniscal bearing relative to the tibial surface (Motion 1).
- The range of variation of $\varphi_{PDF}$ (Δ $\varphi_{PDF}$ ), defined as the overall rotation of the mobile talar component in the working plane, according to the planetary gear model. It represents the plantar-dorsiflexion angle computed by the artificial joint, assuming that point $C_{M}$ follows the path of the instantaneous centre of velocity $C_{A}$ . Maximum plantarflexion and dorsiflexion angle are defined as the maximum value for $\varphi_{PDF}$ , read when $C_{M}$ is imposed to be coincident with E_1_ and E_2_ respectively.
- Anterior posterior motion of the meniscal bearing, defined as the arc length subtended by the angle $\varphi_{1}$ on a circle of radius $r_{TI}$ + $\frac{H}{2}$ , where H is the thickness of the insert. It represents the distance covered by the meniscal bearing when moving from the most posterior to the most anterior configuration.

The behaviour of the ligaments during the analysed motions was also inspected. Origins of both tibiocalcaneal and calcaneofibular ligaments were considered as fixed points; insertions were rigidly rotated from the neutral position to the maximum plantar and dorsi flexion configurations by imposing a rotation of $\varphi_{1}$ around $O_{1}$ followed by the corresponding rotation of $\varphi_{2}$ around $O_{2}$ . Ligament strain ( $\left.\varepsilon \right)$ was computed for each of the n frames and for both calcaneofibular tibiocalcaneal ligaments as the relative change in length $\left(l-l_{N}\right)/l_{N}$ , where l is the distance between ligament insertion and origin points and $l_{N}$ is the corresponding segment length measured in the neutral pose.

As consequence of these strains, the intersection of the ligaments computed according to the rotation imposed on the mobile segment with the FAR implant $C_{I}$ , did not perfectly match with $C_{A}$ . The distance d between $C_{A}$ and $C_{I}$ was considered an index of the incompatibility between the motion allowed by the ankle prosthesis and the physiological motion of the healthy joint.

## 3 Results

### 3.1 Physiological path of the instantaneous centre of rotation during the passive plantar-dorsiflexion motion

**FIGURE 6:**
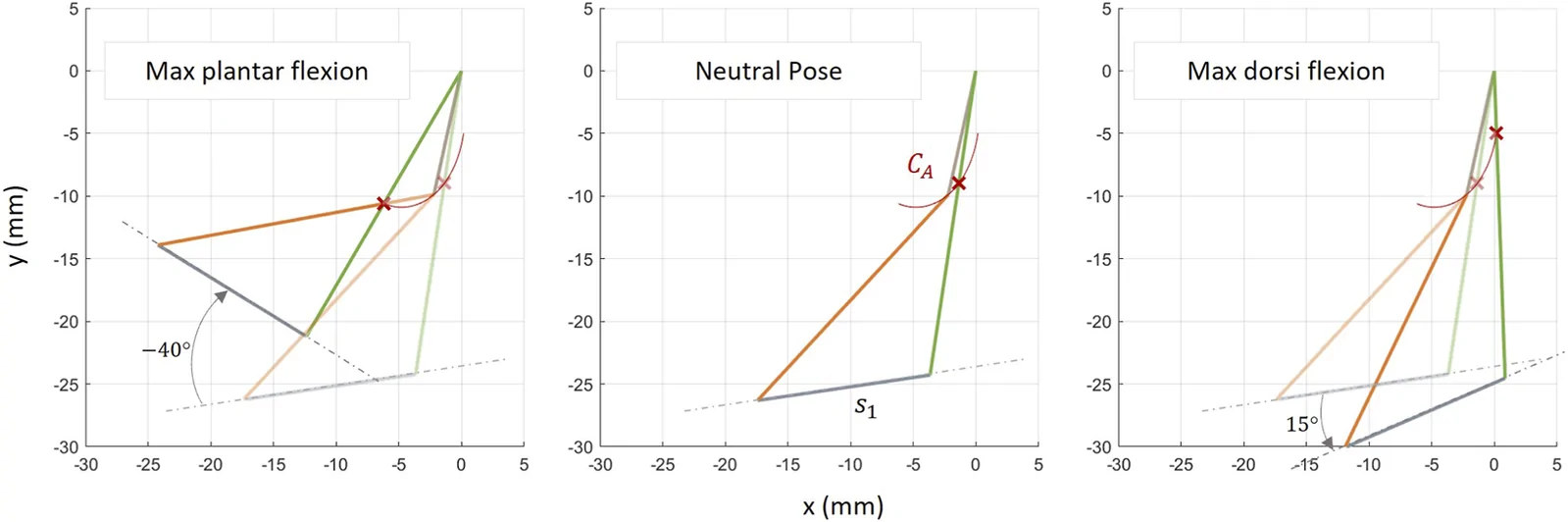
Physiological path of the instantaneous centre of rotation $C_{A}$ obtained with the 4-bar linkage model for the considered plantar-dorsiflexion angles. Configurations corresponding to maximum plantar flexion, neutral position, and maximum dorsiflexion are also shown. The position of $C_{A}$ in these three configurations is marked by a red cross marker.

The path of the instantaneous centre of rotation, $C_{A},$ of the mobile segment $s_{1}$ representing the talus-calcaneus complex with respect to the fixed reference (i.e., the fixed centrode) was obtained using the 4 bar linkage model described in Section 2.2 for a given range of plantar-dorsiflexion angle $\theta_{PDF}$ . Figure 6 shows the discrete data curve describing by the path of $C_{A}$ (in red) computed in the $\bar{n}$ frames, and the orientation of the four segments in three configurations obtained with $\theta_{PDF}=-40{}^{\circ}$ (maximum plantar flexion), $\theta_{PDF}=0{}^{\circ}$ (neutral pose) and $\theta_{PDF}=15{}^{\circ}$ (maximum dorsi flexion). The position of $C_{A}$ in these three configurations is marked by a red cross marker.

### 3.2 Kinematics of the implanted ankle joint

The trends of the gear ratio τ and of the angle $\varphi_{1}$ representing the rotation of the meniscal insert relative to the tibial surface, computed for each of the $\bar{n}$ frames (Equation 11), are shown in Figure 5. The Figure also reports the resulting angle $\varphi_{PDF}$ , which defines the orientation of the talar component relative to its neutral configuration.

The instantaneous gear ratio, τ , ranges from 5.74 at the maximum plantarflexion to 4.50 at the maximum dorsiflexion. Over the same range of motion, $\varphi_{1}$ varies from −2.8° to 0.93°, while $\varphi_{PDF}$ varies from −15.9° to 4.64°. The plantar-dorsiflexion excursion (Δ $\varphi_{PDF}$ ) was 20.54°, corresponding to a Δ $\varphi_{1}$ of 3.74° and an anterior-posterior motion of the meniscal bearing of about 5 mm.

**FIGURE 7:**
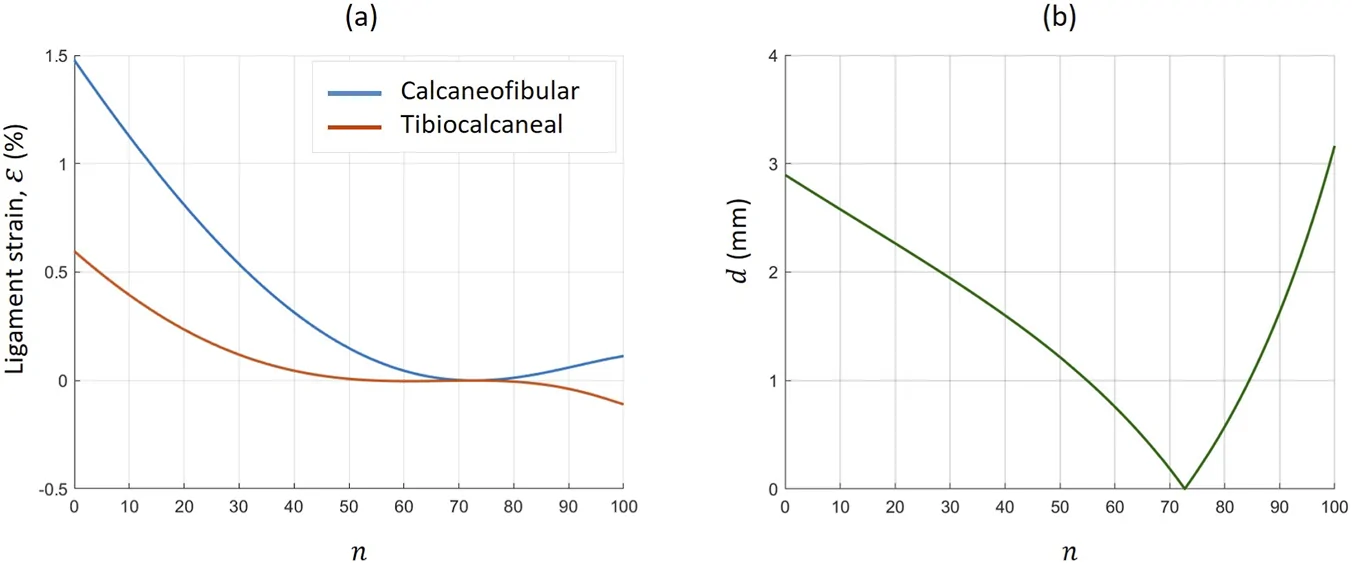
**(a)** Trend of the ligament strain computed for each of the $\bar{n}$ frames and for both calcaneofibular tibiocalcaneal ligaments, expressed as the relative change in length during the simulated movement. **(b)** Trend of the distance d between $C_{A}$ and the point representing the intersection of the ligaments, computed for each of the $\bar{n}$ frames.

The trend of ligament strain variation during the simulated movement is shown in Figure 7a. The maximum relative change in length, although minimal (approximately 1.5%), was observed at the phase of maximum plantarflexion for the calcaneofibular ligament, whereas no variation was detected at the neutral pose.

The intersection of the ligaments $C_{I}$ , calculated according to the rotation imposed on the mobile segment, did not perfectly follow the path of the instantaneous centre of rotation $C_{A}$ , showing a maximum mismatch of 3.16 mm at the maximum dorsiflexion angle. The trend of the distance d between $C_{A}$ and the point representing the intersection of the ligaments, computed during the simulated motion, is shown in Figure 7b.

An animation illustrating the mechanical system developed to study the subject-specific kinematics of the implanted ankle joint is provided in the Supplementary Material (Video 1.mp4).

## 4 Discussion

The aim of this study was to systematically analyse the subject-specific kinematics of an implanted ankle joint, and to investigate the compatibility between the motions allowed by the ankle prosthesis and the physiological motion of the healthy joint. A novel methodology based on the study of an equivalent mechanism, capable of describing the kinematics of the ankle prosthesis in order to more faithfully reproduce the physiological kinematics of the natural joint, was proposed and applied to an implanted FAR prosthesis.

Both physiological and mechanical aspects were taken into account. Starting from a CT-based 3D model of the ankle joint and a well-known geometrical model (Leardini et al., 1999b), a subject-specific 4-bar linkage model was built to estimate the physiological path of the instantaneous centre of rotation of the talus–calcaneus complex relative to the tibia–fibula complex during a prescribed passive motion. In parallel, a systematic analysis of the geometrical and kinematic constraints imposed by the prosthetic surfaces of the FAR implant was conducted. A key innovative aspect of this study was the adoption of an equivalent mechanism, specifically, a planetary gear system with non-circular gears, to reproduce the physiological ankle kinematics with high fidelity while accounting for the geometrical constraints of the implant. Planetary gears were considered suitable for this study due to their intrinsic property of allowing, at least theoretically, pure rolling motion along a predefined curve, which in this specific case corresponded to the path traced by the instantaneous centre of rotation of the talus–calcaneus complex relative to the tibia–fibula complex during the prescribed passive motion. This approach enabled the kinematic analysis of ankle joint motion with the FAR prosthesis.

The overall range of passive plantar-dorsiflexion was estimated to be 20.54°, reduced by 34.46° compared to the 55° achievable by the healthy joint. Indeed, the complete pose the talar segment *s*_1_ of the 4-bar linkage cannot be fully reproduced by a 2 DoFs gear mechanism. For this reason, only the two positional constraints (i.e., ${x_{C_{A}}}^{n}\text{ and }{y_{C_{A}}}^{n}$ giving $C_{A}=C_{M}$ ) were considered in Equation 12 without imposing the condition $\varphi_{PDF}$ = ${\;\theta }_{PDF}$ . To deepen the comparison of the two approaches, the trajectory of $O_{2}$ belonging to segment *s*_1_, was compared with the circular trajectory predicted by implant/gear kinematics obtaining only a partial overlapping (See Video 2.mp4 provided in the Supplementary Material). This difference explains also the non-perfectly rigid behaviour of the ligaments associated to the gear model.

A nearly isometric pattern was observed throughout the motion for both ligaments, with a peak strain of 1.5% recorded in the calcaneofibular ligament during extreme plantarflexion. Despite this, the intersection of the ligaments, computed according to the rotation imposed on the mobile segment, did not perfectly follow the path of the instantaneous centre of rotation $C_{A}$ with a maximum mismatch of 3.16 mm at the maximum dorsiflexion angle. This may be explained by the fact that the designed prosthetic surfaces, whose curvature radii directly influence the gear ratio values, cannot fully restore the physiological motion pattern for this specific subject, given the virtual surgery performed.

It is important to note that the antero–posterior positioning of the implant components must be defined to satisfy the alignment conditions between the three centres of rotation ( $O_{1}$ , $O_{2}$ , and point $C_{A}$ ) in the neutral pose in order to realize the mechanism. This requirement was also highlighted in an early publication on the development of this implant design (Golanó et al., 2010), although no significance was claimed in subsequent studies or surgical procedure descriptions. We argue that deviations from this condition cause the system to adopt a non-balanced configuration, resulting in altered plantarflexion and dorsiflexion angles.

Comparing the results obtained with those reported in the literature, several observations can be made. The 4-bar linkage model built on the subject-specific anatomy was similar in shape to that reported by Leardini et al. (1999b), although the length of each segment was shorter. The path of the instantaneous centre of rotation was also similar to that presented in the original study, although this was achieved with a different imposed range of motion. Regarding the range of motion of the implanted ankle joint and the anteroposterior motion of the meniscal bearing, the results obtained in our study were comparable to those reported in the literature. A prospective study by Giannini (Giannini et al., 2010) analysed 51 patients treated with the BOX prosthesis, observing an average postoperative plantar–dorsiflexion amplitude of 27° and an average anteroposterior meniscal bearing displacement of 4 mm. They reported a statistical correlation between these parameters, which was also observed in our study and can be explained by the geometric features of the implant. A study by Cenni et al. (2012a), which aimed to assess the influence of implant positioning on the range of motion of an ankle implanted with the BOX prosthesis, reported an average plantar–dorsiflexion ROM of 34° measured by postoperative radiographs. The meniscal bearing movement was directly tracked using image markers and averaged 3.6 mm. Comparison between these data and our results is difficult due to a different definition of plantar–dorsiflexion, which in their study is defined as the inclination of the tibial diaphyseal axis relative to the perpendicular to the floor. A more consistent comparison can be made with the data found in a more recent study by Cenni et al. (2012b), in which a 3D kinematic analysis of an ankle joint implanted with the BOX prosthesis was derived from clinical imaging and used to reconstruct the motion patterns of the prosthetic components during various tasks. They reported an average plantar–dorsiflexion ROM of 17.6°, taking into account the relative orientation of the two metallic components of the implant.

It is important to note that the comparison between our results and the clinical data reported in these studies has an inherent limitation, due to the fact that our analyses were restricted to passive plantar–dorsiflexion movements. Other important limitations of our work can be highlighted.

The assigned value of the plantar–dorsiflexion angle, spanning from −40° (plantarflexion) to 15° (dorsiflexion), used to derive the physiological path of the instantaneous centre of rotation and define the range of variation of $\varphi_{1}$ , has an influence on the final results of the proposed methodology. A wider range of variation would also have affected the overall rotation of the mobile talar component relative to the tibial component. However, the chosen range is considered to represent the natural range of ankle flexion; a larger plantar–dorsiflexion range was excluded from the analysis to avoid extreme configurations of the meniscal bearing position that could potentially increase the risk of implant dislocation.

The utmost effort was made in segmenting the anatomical geometries and virtually palpating the anatomical landmarks; however, CT scans are not the most reliable source of information for soft tissues, and a more precise identification of the ligaments could have been achieved using MRI data. This does not affect the considerations made in the study, which can be applied to different anatomical geometries.

Another limitation of the study, which is important to mention, is that the analysis is limited to planar kinematics. A more comprehensive description should be performed in 3D, ideally also including external loads and postoperative soft tissue reconstruction in order to accurately reproduce the complexity of the biomechanical system.

Despite the limitations of the study, the proposed methodology allowed us to draw important conclusions on how to systematically analyse the subject-specific kinematics of an implanted ankle joint and to investigate the compatibility between the motions allowed by the prosthesis and the physiological motion of the healthy joint. The analyses performed provide a basis for further research, which could potentially lead to the definition of optimal strategies for best reproducing physiological joint kinematics in each patient and achieving fully customized implant designs.

## Data Availability

The datasets presented in this article are not readily available because they are part of an ongoing study. Requests to access the datasets should be directed to cristina.curreli@ior.it.

## Supplementary material

DATA SHEET 1 Video 1 description.

DATA SHEET 2 Video 2 description.
