# Supplementary material for: Subject-specific kinematic analysis of a total ankle arthroplasty implant
Source: Front Bioeng Biotechnol. 2026 Aug 28;14:1855705. doi: 10.3389/fbioe.2026.1855705 (PMC13561990; doi:10.3389/fbioe.2026.1855705)
Supplement: Supplementary file 1 [file DataSheet1.docx]

The animation illustrates the mechanical system developed to study the subject-specific kinematics of the implanted ankle joint.

The pink and cyan arcs correspond to the sagittal profiles of the prosthetic component surfaces in contact with the insert (see also Figure 3 and Figure 4). A light green line indicates the minimum thickness of the insert that rotates about the center of the tibial arc. The tibial component (pink curve) is fixed, while the talar component (cyan curve) rotates relative to the meniscal bearing about the center of its arc, proportionally to the rotation of the insert, as defined by the gear ratio (Section 3.2).

It can be observed that, during motion, the center of the talar arc lies on a circumference centered at the tibial arc, with a radius equal to the sum of the talar radius, the tibial radius, and the minimum thickness of the insert. The trajectory of the center of the talar arc is represented by a thin blue line. The center of the tibial arc is not shown due to scaling issues in the animation, but the dashed red line is directed along the radius.

The physiological path of the instantaneous center of rotation during passive plantar–dorsiflexion, $C_{A}$, obtained from the four-bar mechanism analysis (Section 3.1), is shown in red, with a cross marker indicating each of the *n* frames of the analysis (see also Figure 5 and Figure 6).

The ligaments are also represented (orange and green dashed lines). Their origins are fixed, while their insertions move rigidly with the talar component (see Section 2.6). The intersection point of the ligaments (black dot) calculated according to the rotation imposed on the mobile segment, did not perfectly follow the path of the instantaneous centre of rotation $C_{A}$ (Section 3.2). The red cross marker and the black dot almost overlap only for a limited number of frames around the neutral pose, where ligament strain tends toward zero.
