# Supplementary material for: Subject-specific kinematic analysis of a total ankle arthroplasty implant
Source: Front Bioeng Biotechnol. 2026 Aug 28;14:1855705. doi: 10.3389/fbioe.2026.1855705 (PMC13561990; doi:10.3389/fbioe.2026.1855705)
Supplement: Supplementary file 2 [file DataSheet2.docx]

The animation illustrates the physiological path of the instantaneous centre of rotation $C_{A}$ obtained with the four-bar linkage model for the considered plantar-dorsiflexion angles (Section 3.1). The position of $C_{A}$ is marked by a red cross marker.

The trajectory of the center of the talar arc, $O_{2}$, considered as belonging to segment $s_{1}$, is represented by the gray point, whereas the blue curve shows the circular trajectory predicted by the implant/gear kinematics (see also the blue trajectory of the talar arc center in Animation 1). The mismatch between these trajectories provides further evidence that the analyzed TAA design was unable to fully reproduce the physiological kinematics of the healthy joint model, as predicted by the four-bar linkage model, for the subject and virtual surgery considered (Section 4).
